# Sample size in cluster randomised trials with unequal clusters

**DOI:** 10.1186/1745-6215-12-S1-A25

**Published:** 2011-12-13

**Authors:** Ivana Holloway, Amanda Farrin

**Affiliations:** 1 Clinical Trials Research Unit (CTRU), University of Leeds, Leeds, UK

## Objectives

The aim of RCTs is to obtain unbiased estimates of treatment effects to answer the question of interest. In a cluster randomised trial (CRT), maximum statistical efficiency is obtained if an equal sized sample from each cluster is selected. In practice, the ability to achieve equal cluster size is an exception rather than the norm. Two CRTs run by the Leeds CTRU; TRACS (Training Caregivers After Stroke) and LoTS Care (Longer Term Stroke Care) are used to demonstrate how this issue can be dealt with practically.

## Methods and results

Unbalanced cluster size decreases the statistical power in CRTs. Even if the original sample size calculation considers clustering, this sample size is underestimated in the case of unequal cluster size. A common reason for ignoring variability of cluster size is the lack of appropriate, easily usable sample size calculation formulae.

In the TRACS trial, all centres were randomised at the same time and unequal cluster size was not anticipated. However, differences in recruitment rate, a higher than expected loss to follow-up and varying by centre occurred. Theoretically, including more clusters, each recruiting the same number of patients, would be an optimal solution. In practice, due to time, logistics and budget constraints, the number of centres was fixed, so overall more participants were recruited and the maximum cluster size was capped. In the LoTS Care trial, imbalances were expected, because centres were randomised in two phases and the overall recruitment period was fixed.

In both trials, we re-assessed sample size calculations and studied the effect of conservative, typical and extreme scenarios in terms of cluster size on the statistical power. In the calculations, various values of drop-out rate, design effect and coefficient of variation were considered and the effect on statistical power was calculated. Using the most conservative estimates, the overall power dropped by 2-3% when compared to calculation of the power based on equal cluster size. For both trials, statistical power based on equal cluster size was estimated to be 90%; so in the presence of unequal cluster size, power above 80% was preserved.

## Conclusions

Using TRACS and LoTS Care trials as examples, we have demonstrated the importance of incorporating unequal cluster sizes into calculations of robust sample size for CRTs.

